# Health trajectories of individuals who quit active religious attendance: analysis of four prospective cohort studies in the United States

**DOI:** 10.1007/s00127-023-02497-x

**Published:** 2023-06-07

**Authors:** Markus Jokela, Michael Laakasuo

**Affiliations:** Department of Psychology and Logopedics, University of Helsinki, Haartmaninkatu 3, 00290 Helsinki, Finland

**Keywords:** Religiosity, Alcohol, Smoking, Health, Religious attendance, Longitudinal

## Abstract

**Purpose:**

To examine whether trajectories of health (depressive symptoms, psychological wellbeing, self-rated health, and body mass index) and health behaviors (smoking, heavy alcohol consumption, physical inactivity, and cannabis use) changed for individuals who first reported at least monthly religious attendance and then in subsequent study waves reported no active religious attendance.

**Methods:**

Data were from four cohort studies from the United States collected between 1996 and 2018: National Longitudinal Survey of 1997 (NLSY1997); National Longitudinal Survey of Young Adults (NLSY-YA); Transition to Adulthood Supplement of the Panel Study of Income Dynamics (PSID-TA); and Health and Retirement Study (HRS) with a total n = 6592 individuals and 37,743 person-observations.

**Results:**

None of the 10-year trajectories of health or health behaviors changed for the worse after the change from active to inactive religious attendance. Instead, the adverse trends were observed already during the time of active religious attendance.

**Conclusion:**

These results suggest that religious disengagement is a correlate—not a cause—of a life course characterized by poorer health and health behaviors. The religious decline caused by people leaving their religion is unlikely to influence population health.

**Supplementary Information:**

The online version contains supplementary material available at 10.1007/s00127-023-02497-x.

## Introduction

Religious affiliation and attendance have been declining in the United States. Between 2007 and 2019, the proportion of people reporting at least monthly religious attendance fell from 54 to 45% [1]. Religious attendance has been associated with better health, such as higher self-rated health [2] and lower depressive symptoms [3], and with healthier habits, such as lower alcohol consumption and lower probability of smoking [4–7]. If religious attendance is a causal factor that promotes better health and healthy behaviors [8], then the decline in religious attendance might contribute to worsening population health in the coming years.

Surprisingly few studies of religiosity and health have probed the issue of causality beyond longitudinal studies with baseline adjustments [3, 9]. Longitudinal studies are more informative than cross-sectional studies, but studies with just two measurement times cannot fully capture the long-term health trajectories that could offer further evidence for causality. Additional research designs are needed. Some studies have adjusted for one measurement time prior to baseline [10, 11], which helps to mitigate reverse causation. Sibling analyses have shown associations of religiosity with alcohol use [5, 6] and psychological distress [12] to be partly or completely confounded by familial factors (i.e., shared family background and/or shared genetics). But sibling analysis only takes into account familial confounding, so these studies cannot provide strong evidence to support or refute causal interpretations.

Other study designs can add important pieces of evidence to evaluate the plausibility of causal claims, even if the ultimate proof for causality remains elusive when controlled experiments are not feasible [13–16]. *Temporal ordering* between variables is an uncontroversial criterion for causality, as the cause should precede the effect. Causality can also be inferred on the basis of *reversibility*: if a cause is removed, one should observe a change in the outcome as compared to a time when the cause was present (this is not a necessary criterion, of course, because some causes can be irreversible). In the case of declining religious attendance and health, we can evaluate the plausibility of causal effects with longitudinal observational data by examining how people’s health status and health behaviors change after the person changes from active to inactive religious attendance. This is the key question for the impact of religious decline on population health: how large changes in health and health behaviors can we expect to occur when people give up active religious attendance?

The present study focused on individuals who first reported active religious attendance but then became inactive. We used repeated measurements of health and health behaviors taken up to 10 years before and after the change in religious attendance. This approach provides a more comprehensive perspective on the temporal associations than the longitudinal studies with only baseline and single follow-up. Assuming that religious attendance is a causal protective factor, we hypothesized that the participants’ health status and health behaviors would start to decline, or decline more steeply, after they quit frequent religious attendance. The weight of evidence would tip against causality if the health trajectories didn’t change with the change in religious attendance, or if adverse health trajectories were to precede the change in religious attendance. To bolster the robustness of the analysis, we pooled data from three cohort studies of young adults and one cohort study of older adults.

## Participants

Participants were from four prospective cohort studies that have collected data annually or biennially: (1) The National Longitudinal Survey of Youth 1997 (NLSY1997) consists of a nationally representative sample of 8,984 men and women born during the years 1980 through 1984 and living in the United States at the time of the initial survey in 1997. Interviews were conducted annually from 1997 to 2011 and biennially since then; (2) the National Longitudinal Survey of Young Adults (NLSY-YA) began in 1986 as an ongoing survey of children born to the female respondents of the 1979 National Longitudinal Survey of Youth; (3) the Transition to Adulthood sample of the Panel Study of Income Dynamics (PSID-TA) is a study of the children of the original PSID sample of roughly 18,000 people in 5,000 households, which includes a nationally representative sample and an oversample of low-income families. The PSID-TA started in 2005 and data collection is carried out every two years; and (4) the Health and Retirement Study (HRS) is a nationally representative longitudinal study of more than 30,000 individuals representing the U.S. population older than 50 years. Telephone or in-person interviews are conducted every 2 years. Details of the cohorts are provided in Online Supplementary Material. Only participants aged 18 years or older were included in the analysis.

### Measures

Religious attendance was reported as the frequency of attending religious services in the last year (NLSY97, NLSY-YA, and HRS) or as the frequency of attending religious services reported as times per year/month/week/day (PSID-TA). Health behaviors were assessed with four self-reported indicators: smoking (current smoker vs. not), heavy alcohol consumption (14 or more weekly alcohol units for men, 7 or more for women), physical inactivity (less than weekly moderate or vigorous physical activity; measures varied), and cannabis use (whether or not had used in last 12 months). Health status was assessed with four self-reported indicators: depressive symptoms (CES-D), psychological wellbeing (Ryff’s psychological wellbeing scale), self-rated general health (1 = poor, 5 = excellent), and body mass index (calculated from self-reported height and weight as kg/m^2^). NLSY97 did not have data on physical activity; HRS did not assess cannabis use; psychological wellbeing was assessed only in HRS and PSID-TA. Details of the measures are described in Online Supplementary Material.

### Statistical analysis

The analysis proceeded as follows: Follow-up time for each participant was determined with an index study wave, and the eligible study waves preceding and following this index wave. The index wave was selected so that the participant (a) reported monthly or weekly religious attendance at the index wave, (b) reported monthly or weekly religious attendance at least in the preceding wave, (c) reported no or less than monthly religious attendance in the wave following the index wave. The index wave was selected for each participant so that it maximized the total available follow-up time for the participant by extending the follow-up backwards in time as far as the participant reported monthly or weekly religious attendance and forwards in time as far as the participant reported no or less than monthly religious attendance. The end result for each participant was the participant’s longest possible follow-up time that comprised an uninterrupted series of active religious attendance followed by an uninterrupted series of inactive religious attendance. For example, if a participant reported religious attendance in 8 study waves as “no, yes, yes, yes, no, no, no, and yes” the participant would have been included with 6 study waves of “yes, yes, yes, no, no, and no” with the first observation of “no attendance” and the last observation of “yes attendance” being excluded from the analysis because they interrupted the follow-up periods of continuous attendance before index wave and continuous non-attendance after index wave. Missing data also interrupted the extension of the follow-up period.

We then fitted multilevel linear regressions (continuous outcomes) and logistic regressions (dichotomous outcomes) in which the outcome was predicted by categorically coded follow-up time (as described above, with index wave as the reference category), age, gender, and self-reported race/ethnicity; in some cases the multilevel model would not converge in which case we used ordinary regression with robust estimation of standard errors to take into account the non-independence of the person-observations. The models were first fitted separately in each dataset and then the coefficients of follow-up time were pooled with random-effects meta-analysis. We then plotted the coefficients against follow-up time, supplementing the scatterplots with linear trends across the meta-analytic coefficients before and after the index wave. This was done with linear regression in which the coefficients were predicted by linear follow-up time, dichotomous indicator of follow-up time before/after the index, and their interaction effect, which allowed the linear trend to be different before and after the index wave. Inverse variance weighting was applied in these regression models to take into account differences in the accuracy of the estimates. The index wave did not have a standard error because it was the reference group, so we imputed the missing standard error based on the sample size of the follow-up time categories (the association between sample size and standard error was almost linear).

In addition to the categorical coding of follow-up time just described, we also fitted the health trajectories using follow-up time as a continuous variable in each cohort-specific analysis by applying piecewise multilevel regression [17] to make sure any changes in trajectories did not go undetected because of using categorized follow-up time. The piecewise regression model takes the form: Y_ij_ = β_0i_ + β_1i_t_ij_d_ij_ + β_2i_t_ij_(1 − d_ij_) + e_ij_, where Y_ij_ is the outcome for person i at time j; β_0i_ is the intercept for person i; t_ij_ is the follow-up time so that t_ij_ = 0 at the person’s index wave, t_ij_ gets negative values (in years) before the index wave and positive values after the index wave; d_ij_ is a dichotomous indicator indicating the time period before vs after the index wave; β_1i_ and β_2i_ are regression coefficients for the trajectories; and e_ij_ is the error term.

Three sensitivity analyses were also included. First, we examined whether the results were different when using a comparison of weekly vs. less frequent religious attendance (instead of at least monthly vs. less often). Second, we fitted the regression models with fixed-effect estimation to remove stable between-individual differences in the average level of the outcome. The fixed-effect estimation thus applied to the passage of time, not to a time-varying indicator of religious attendance. The analyses were adjusted for age, gender, race/ethnicity, education (1 = less than high school, 2 = high school, 3 = college or higher), and time-varying indicator of marital status (1 = not married, 2 = married, 3 = divorced/separated, 4 = widowed). Third, in order to safeguard against any methodological artefacts that might have been introduced by our approach, we examined what the shape of the trajectories would have been regardless of religious attendance by selecting the index wave randomly for all available participants (i.e., whether or not they attended or not) and running the main analysis with these samples; this last analysis should not have produced any meaningful differences before vs. after the index wave.

Finally, to complement to the main analysis of religious disengagement, we created another indicator for religious attendance that reversed the direction of change, that is, starting with participants who did not report religious attendance and then reported active religious attendance after the index wave. The analysis procedure was otherwise exactly the same as described above.

Altogether, the NLSY1997 had 8769 participants with at least one measurement time of religious attendance; 6173 participants who at least once reported at least monthly religious attendance; 4825 participants who had at least one measurement before and one after a study wave with active religious attendance; and 2237 participants with at least one measurement with active attendance before and at least one measurement time of inactive attendance after the index wave. The corresponding numbers were 8080, 5576, 3736, and 1262 for NLSY-YA; 3924, 2223, 1001, and 298 for PSID-TA; and 18,467, 12,810, 10,876, and 2795 for HRS. The intraclass correlation of religious attendance over time in the four cohort studies was 0.54 (NLSY1997), 0.52 (NLSY-YA), 0.62 (PSID-TA), and 0.73 (HRS).

## Results

Table 1 reports the summary descriptive statistics and Supplementary Table 1 presents the full descriptive statistics by study wave.

**Table 1:** Descriptive statistics of the analytic samples

	NLSY97	NLSY-YA	PSID-TA	HRS
Categorical variables^†^				
Women	8807 (55.4)	2983 (53.0)	623 (53.2)	9776 (64.9)
Race/ethnicity				
Non-Black, non-Hispanic	7186 (45.2)	1952 (34.7)	490 (41.8)	10,230 (67.9)
Black/African-American	5038 (31.7)	1276 (22.7)	581 (49.6)	2602 (17.3)
Hispanic	3515 (22.1)	2398 (42.6)	–	1926 (12.8)
Other	150 (0.9)	–	100 (8.5)	299 (2.0)
Religious attendance				
Never	1763 (11.1)	572 (10.2)	136 (11.6)	2136 (14.2)
Rarely	5195 (32.7)	1586 (28.2)	303 (25.9)	3054 (20.3)
Monthly	4401 (27.7)	2140 (38.0)	450 (38.4)	3688 (24.5)
Weekly	4530 (28.5)	1328 (23.6)	282 (24.1)	6179 (41.0)
Smoking	3552 (23.0)	1200 (21.5)	248 (21.2)	1613 (12.2)
Heavy alcohol consumption	1709 (11.2)	349 (7.1)	63 (5.4)	164 (1.3)
Physical inactivity	–	1152 (23.7)	566 (48.3)	6932 (46.1)
Cannabis use	1552 (10.2)	873 (15.8)	323 (27.6)	–
Continuous variables^‡^				
Depressive symptoms*	0.9 (0.5)	0.6 (0.6)	0.6 (0.5)	1.7 (2.1)
Psychological wellbeing**	–	–	5.1 (0.8)	4.4 (0.9)
Self-rated health (1 to 5)	3.9 (0.9)	3.8 (1)	3.8 (0.9)	2.9 (1.1)
Body mass index (kg/m^2^)	27 (6.3)	26.9 (6.1)	26.9 (5.9)	28.2 (6.3)
Age (years)	24.6 (3.8)	23.7 (3.6)	22.1 (2.6)	72.6 (11.1)
Waves before index wave	3.6 (2.8)	1.9 (1.0)	1.5 (0.7)	2.9 (1.6)
Waves after index wave	4.1 (3.2)	1.9 (1.1)	1.6 (0.8)	2.1 (1.3)
n (person-observations)	15,889	5626	1171	15,057
n (persons)	2237	1262	298	2795

^†^Values are numbers (and percentages) of person-observations

^‡^Values are means and standard deviations

*Reported as mean score (response scale from 0 to 3) except for HRS as sum score of 8 dichotomous (yes, no) items

**Reported as mean score (response scale from 1 to 6)

**Fig. 1:**
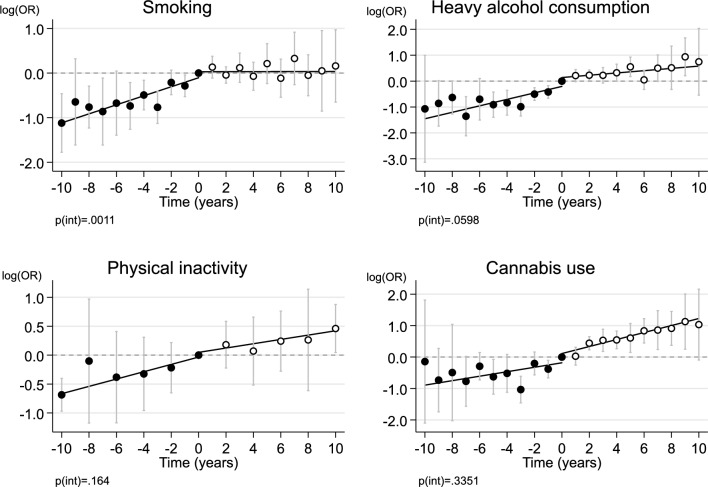
Log odds ratios (logOR) for health behaviors before and after the change from active to inactive religious attendance (monthly or more vs. less), pooled across the four cohort studies with random-effects meta-analysis. The reference category is zero, the index wave of the participant’s last measurement time of active religious attendance during the follow-up time. Error bars are 95% confidence intervals. p(int) = statistical significance for the difference in fitted lines before vs after index wave. Maximum n = 37,721 person-observations of 6592 individuals

Figure 1 shows the meta-analytic estimates between religious attendance and health behaviors before and after the change from active to inactive religious attendance. The estimates for even years are pooled across all cohorts, the estimates for odd years are only from NLSY97. Heavy alcohol consumption, physical inactivity, and cannabis use increased throughout the follow-up period but did not change at the index wave when the change from active to inactive religious attendance happened. Only the trajectory for smoking changed at the index wave by becoming flat after having increased before the index wave (see Supplementary Figs. 1 to 4 for cohort-specific associations). The direction of this change was in the opposite direction what we hypothesized. The piecewise regression method yielded similar results to the categorical analysis described above: smoking increased before and did not change after the index wave, but there were no other changes at the index wave (Table 2 and Supplementary Figs. 5 to 8). These associations were similar but less precisely estimated when using fixed-effect regression that was based only on the within-individual change (Supplementary Fig. 9 and Supplementary Table 2). The results remained unchanged when religious attendance was categorized as weekly vs less frequent attendance rather than at least monthly vs less frequent, which was used in the main analysis (Supplementary Fig. 10 and Supplementary Table 2).

**Table 2:** Health trajectories before and after the change from active to inactive religious attendance

Variable	Before	After	p (int)
Smoking*	0.11 (0.04, 0.17)	− 0.01 (− 0.07, 0.05)	0.01
Heavy alcohol use*	0.17 (0.07, 0.27)	0.05 (− 0.03, 0.13)	0.07
Physical inactivity*	0.07 (− 0.05, 0.20)	0.02 (− 0.09, 0.13)	0.58
Cannabis use*	0.09 (0.01, 0.17)	0.14 (0.10, 0.19)	0.25
Depressive symptoms^†^	0.01 (− 0.01, 0.03)	0.01 (− 0.02, 0.03)	0.82
Psychological wellbeing^†^	0.01 (− 0.03, 0.05)	− 0.01 (− 0.03, 0.00)	0.21
Self-rated health^†^	− 0.02 (− 0.04, 0.01)	− 0.02 (− 0.05, 0.00)	0.84
Body mass index^†^	0.12 (0.10, 0.15)	0.08 (0.05, 0.10)	0.01

Values are meta-analytic estimates for regression coefficients that indicate the slope of trajectories (difference in the outcome per 1 year) from piecewise linear or logistic regression models, adjusted for age, sex, race/ethnicity, education, and marital status (maximum n = 37,721 person-observations of 6592 individuals)

p(int) = statisticial significance of the difference between before vs after

*Models fitted with logistic regression

^†^Models fitted with linear regression

**Fig. 2:**
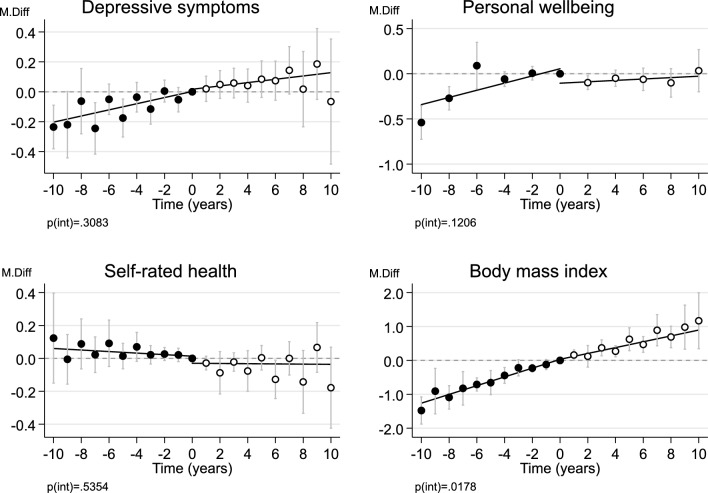
Mean differences (M.Diff) in health outcomes before and after the change from active to inactive religious attendance (at least monthly vs. less often), pooled across the four cohort studies with random-effects meta-analysis. The reference category is zero, the index wave of the participant’s last measurement time of active religious attendance during the follow-up time. Error bars are 95% confidence intervals. p(int) = statistical significance for the difference in fitted lines before vs after index wave. Values are standardized scores (standard deviation = 1) for depressive symptoms and psychological wellbeing, and non-standardized scores for self-rated health and body mass index. Maximum n = 37,721 person-observations of 6,592 individuals

Figure 2 shows the associations for health indicators. Depressive symptoms and BMI increased, and self-rated health slightly decreased, during the follow-up period. Only BMI showed a change at the index wave so that the increase became less steep after the index wave (see Supplementary Figs. 11 to 14 for cohort-specific results), which again was the opposite to what we hypothesized. The piecewise regressions provided similar results to those fitted with categorical time points (Table 2 and Supplementary Figs. 15 to 18). None of the changes at index wave were significant in the fixed-effect regressions (Supplementary Fig. 19 and Supplementary Table 2) or when comparing between weekly vs less than weekly attendance (Supplementary Fig. 20 and Supplementary Table 2).

To test whether the results reported above could have arisen spuriously, we re-run the analysis by selecting the index wave at random for each participant in the complete cohort studies: health behaviors showed no clear trends whereas self-rated health decreased, and BMI and psychological wellbeing increased (Supplementary Figs. 21 and 22). There were no changes in the trajectories at the index wave.

Supplementary Fig. 23 shows the reverse temporal associations from inactive to active religious attendance (see Supplementary Figs. 24 to 31 for cohort-specific associations). Smoking declined more steeply after the index wave, that is, after the participants started active religious attendance after having been inactive. Self-rated health declined before but not after the index wave. These results were also observed with the piecewise regression models (Supplementary Table 3). The fixed-effect regressions were similar but less precise (Supplementary Table 3, Supplementary Fig. 32), and the results were quite similar also with weekly vs. less frequent religious attendance (Supplementary Table 3, Supplementary Fig. 33).

## Discussion

The goal of this study was to provide indirect evidence regarding causality in the association between declining religious attendance and health. If active religious attendance did improve health and promote better health behaviors, we would expect people’s health to get worse after they quit active religious attendance. Data from four prospective cohort studies provided little if any evidence to support this hypothesis.

Heavy alcohol consumption, smoking, physical inactivity, cannabis use, and body mass index increased, and self-rated health decreased, already in the 10-year follow-up period before the change from active to inactive religious attendance, and these trajectories did not become steeper in the following years of inactive religious attendance. These results lend support for a non-causal interpretation in which religious attendance is a correlate—not a cause—of poor health-behavior trajectories. It seems plausible that people who are on a life-course trajectory characterized by poorer health behaviors are also more likely to quit active religious attendance. Crucially, this non-causal interpretation appears more plausible than a bidirectional causal association between religious attendance and health, which has been suggested by some previous studies [11]. If religious attendance had been a causal protective factor, poorer health and health behaviors should have worsened even more after the participants gave up religious attendance, but this was not the case. This speaks against the hypothesis of bidirectional causality.

Smoking increased in the 10 years before the change from active to inactive religious attendance but remained flat afterwards. Similarly, the increase in BMI became less steep, and heavy alcohol consumption showed slightly (but not significantly) more gradual increase after versus before the change. These patterns were unexpected, and this study design was not equipped with measures to examine these trends with sufficient depth. It is possible that religious engagement induces psychological distress (and associated poor health behaviors) in some individuals [18], and that this distress is relieved after giving up religious engagement. More specific measures of religiosity would be required to test this hypothesis. For the other direction of change in religious attendance—from inactive to active—there was evidence for decreasing smoking and slightly improving self-rated health after starting active religious attendance.

None of the above is to say that people who attend religious services don’t have better health and health behaviors than those not attending. Previous studies have also reported poorer health among those who have left religion [19–21], but these retrospective studies have not been able to determine temporal precedence between health and leaving religion. The current longitudinal findings show that the unhealthy developmental trajectories were observed already several years before becoming religiously inactive, that is, during the time the participants still reported at least monthly religious attendance. It appears more likely that the poorer health among those who have left religion [19–21] represents adverse health trajectories preceding religious disaffiliation—and not its consequences.

It can be argued that most causal questions are meaningful only when considered against the backdrop of an intervention—even an imagined intervention that would be impossible to carry out in the real world [13]. This helps to clarify the specific causal claim being evaluated. The present study was motivated by the question of whether declining religious attendance will lead to deteriorating population health, and so the analysis focused on changes in health and health behaviors among individuals who initially engaged in religious activities but then became disengaged. It is safe to assume that most people who quit religious attendance in the United States do so voluntarily, without external coercion, so the study design should be suitable for answering the question concerning religious decline due to people leaving their religion. But the study design did not address all aspects of religious decline, as religious engagement may also decrease due to birth-cohort effects by which more recently born individuals are less religious to begin with [22]. It is unclear whether the present conclusions extend to birth-cohort effects, as different social and causal mechanisms might be involved. The focus on changing religious attendance also means that the participants might not have been representative of all religiously active and inactive individuals; study designs attempting to address specific causal claims often need to rely on non-representative sampling [23]. Other study designs would be needed to assess causality of religiousness among those who remain either religiously active or inactive throughout their life.

In 2018–2019, 65% of Americans identified themselves as Christians and 26% as religiously unaffiliated, which leaves only 9% for all the other major religions, such as Hinduism, Islam, and Buddhism [1]. The present study did not have large enough samples to examine differences between religions, so it is uncertain whether the present results generalize to other religions besides Christianity. Moreover, some Christian denominations (e.g., Mormons and Jehovah’s Witnesses) within the United States often have stricter social norms that might influence health behaviors more strongly than what was observed in the present study [20], but these denominations cover only a small proportion of the population.

It should also be emphasized that the current study focused on religious attendance and did not address religious disaffiliation, that is, the increasing prevalence of the religiously unaffiliated, or any other measures of religiosity/spirituality. By itself, religious affiliation is unlikely to have causal effects on health, whereas religious attendance has been shown to be the indicator of religiosity that has the strongest correlations with health, or at least with depressive symptoms [3]. Religious attendance has also been shown to account for the health correlates of religious affiliation [21], which further justifies the use of religious attendance as the measure of religiousness when examining health outcomes. Health changes associated with changes in other indicators of religiosity/spirituality should nevertheless be examined with similar study designs. People may exaggerate their frequency of religious attendance due to social desirability bias, which may have introduced measurement inaccuracy in our analysis [24, 25].

In sum, the present study suggests that the declining rates of religious attendance are unlikely to have a major influence on population health: most of the adverse health correlates of religious non-attendance were observed already many years before the participants gave up active religious attendance, and the change from active to inactive religious attendance did not change the trajectories of health or health behaviors for the worse. It is more likely that religious disengagement is an expression of broader life-course trajectory accompanied by poorer health and health behaviors.

### Supplementary Information

Below is the link to the electronic supplementary material.

Supplementary file1 (DOCX 1966 KB)

## Data Availability

The data are available for download at the study websites, which are referenced in Supplementary Material.
