# Supplementary material for: Health trajectories of individuals who quit active religious attendance: analysis of four prospective cohort studies in the United States
Source: Soc Psychiatry Psychiatr Epidemiol. 2023 Jun 7;59(5):871–8. doi: 10.1007/s00127-023-02497-x (PMC11087366; doi:10.1007/s00127-023-02497-x)
Supplement: Supplementary file 1 — Supplementary file1 (DOCX 1966 KB) [file 127_2023_2497_MOESM1_ESM.docx]

**Health trajectories among individuals who quit active religious attendance: Analysis of four prospective cohort studies in the United States**

Online Supplementary Material

**Contents**

Description of cohort studies ………………………………………………………… 2

Supplementary Tables …………………………………………………………………6

Supplementary Figures ………………………………………………………………..12

Health and Retirement Study (HRS)

The HRS is a nationally representative longitudinal study of more than 30,000 individuals representing the U.S. population older than 50 years. Telephone or in-person interviews are conducted every 2 years, administered under the NIA and the University of Michigan’s Institute for Social Research. As of 1998, the HRS consists of 4 sources of data collection: (1) The original HRS began as two distinct surveys that were merged in 1998. The original HRS was initially administered in 1992 to a nationally representative sample of Americans born in the years 1931 through 1941. In the case of married couples, both spouses (including spouses who were younger than 51 or older than 61) were also interviewed; (2) The second survey, originally referred to as the Study of Assets and Health Dynamics Among the Oldest Old (AHEAD), was first administered in 1993 to a nationally representative sample of Americans born in 1923 or earlier (n=8,000) and merged with the HRS in 1998. In the case of married couples, interviews were conducted with both spouses; (3) In 1998, a subsample of individuals born between 1924 and 1930, referred to as Children of the Depression Age (CODA) was added to HRS; (4) Another subsample consisting of people born between 1942 and 1947 (War Baby cohort) was added to replenish the sample of people in their early 50s as the original HRS cohort aged. The Health Sciences Institutional Review Board at the University of Michigan approved the HRS.

**Measures:** Religious attendance was assessed with the question “About how often have you attended religious services during the past year?” with the response options of 1=Not at all, 2=One or more times a year, 3=Two or three times a month, 4=Once a week, 5=More than once a week. Smoking was coded as current smoker vs. not. Heavy alcohol consumption was determined based on self-reported weekly intake and defined as 14 units or more men and 7 or more units for women. Physical inactivity was assessed based on self-reported frequency of moderate and vigorous physical activity, both items rated a scale from 0 to 4 (0=hardly ever or never; 1=one to three times a month; 2=once a week; 3=more than once a week; 4=every day) and defined as no moderate or vigorous activity (i.e., response of zero to both questions). Depressive symptoms were assessed with 8 items of the CESD (response scale no/yes). Personal wellbeing was assessed with a 21-item version of Ryff’s personal wellbeing scale. Self-rated general health was reported on a 5-point scale (1=poor, 5=excellent). Body mass index was calculated from self-reported height and weight.

Study website: <http://hrsonline.isr.umich.edu>

Acknowledgements: The HRS (Health and Retirement Study) is sponsored by the National Institute on Aging (grant number NIA U01AG009740) and is conducted by the University of Michigan. The Health and Retirement Study public use dataset (Ann Arbor, MI, 2016) is produced and distributed by the University of Michigan.

National Longitudinal Survey of Young Adults (NLSY-YA)

The National Longitudinal Survey of Youth 1979 (NLSY79) is a nationally representative sample of 12,686 young men and women born during the years 1957 through 1964 and living in the United States when the survey began. In 1986, a separate survey of all children born to NLSY79 female respondents began. The child survey includes assessments of each child as well as additional demographic and development information collected from either the mother or child. For children aged 10 and older, information has been collected from the children biennially since 1988 on a variety of factors including child-parent interaction, attitudes toward schooling, dating and friendship patterns, religious attendance, health, substance use, and home responsibilities. Biennially (since 1994), children ages 15 and older complete interviews modeled on the NLSY79 questionnaire. Information collected includes their schooling, training, work experiences and expectations, health, dating, fertility and marital histories, and household composition. Starting in 2016, NLSY79 children age 12 and older were included in the Young Adult data collection.

**Measures:** Religious attendance was assessed with the question “In the past year about how often have you attended religious services?” with response options of 1=Not at all, 2=Several times a year or less, 3=About once a month, 4=Two or three times a month, 5=About once a week, 6=More than once a week. Smoking was coded as current smoker vs. not. Heavy alcohol consumption was determined based on self-reported weekly intake and defined as 14 units or more men and 7 or more units for women. Physical inactivity was assessed based on self-reported frequency of doing strenuous exercise at least for 15min; moderate exercise at least for 15min; and strength training exercise at least for 15min (1=zero times per week, 2=1 time per week, 3=2-3 times per week, 4=4-5 times per week, 5=six or more times a week) and defined as doing any of the three exercise types more than once a week. Cannabis use was assessed as any use within last year. Depressive symptoms were assessed with 7 items of the CESD (4-point response scale). Self-rated general health was reported on a 5-point scale (1=poor, 5=excellent). Body mass index was calculated from self-reported height and weight.

Study website: <https://www.bls.gov/nls/>

**Acknowledgements:** The NLSY79 survey is sponsored and directed by the U.S. Bureau of Labor Statistics and managed by the Center for Human Resource Research (CHRR) at The Ohio State University. Interviews are conducted by the National Opinion Research Center (NORC) at the University of Chicago. The Children of the NLSY79 survey is sponsored and directed by the U.S. Bureau of Labor Statistics and the National Institute for Child Health and Human Development. The survey is managed by the Center for Human Resource Research (CHRR) at The Ohio State University and interviews are conducted by the National Opinion Research Center (NORC) at the University of Chicago.

National Longitudinal Survey of Youth 1997

The NLSY97 consists of a nationally representative sample of 8,984 men and women born during the years 1980 through 1984 and living in the United States at the time of the initial survey in 1997. Participants were ages 12 to 16 as of December 31, 1996. Interviews were conducted annually from 1997 to 2011 and biennially since then. The NLSY97 collects information on respondents’ labor market behavior and educational experiences. The survey also includes data on the youths’ family and community backgrounds to help researchers assess the impact of schooling and other environmental factors on these labor market entrants.

**Measures:** Religious attendance was assessed with the question ”In the past 12 months, how often have you attended a worship service like a church or synagogue service, or a service at a mosque?” with response options of 1=Never, 2=Once or twice, 3=Less than once a month, 4=About once a month, 5=About twice a month, 6=About once a week, 7=Several times a week, 8=Everyday. Smoking was coded as current smoker vs. not. Heavy alcohol consumption was determined based on self-reported weekly intake and defined as 14 units or more men and 7 or more units for women. Cannabis use was assessed as any use within last year. Depressive symptoms were assessed with 5 items of the CESD (4-point response scale). Self-rated general health was reported on a 5-point scale (1=poor, 5=excellent). Body mass index was calculated from self-reported height and weight.

Study website: <https://www.bls.gov/nls/>

**Acknowledgements:** The NLSY97 survey is sponsored and directed by the U.S. Bureau of Labor Statistics and and managed by the Center for Human Resource Research (CHRR) at The Ohio State University. Interviews are conducted by the National Opinion Research Center (NORC) at the University of Chicago.

Panel Study of Income Dynamics, Transitionin to Adulthood Supplement (PSID-TA)

The original PSID sample of roughly 18,000 people in 5,000 households consisted of a nationally representative sample and an oversample of low- income families. The Child Development Supplement (CDS) collects extensive data on children and adolescents and their primary caregivers in PSID families. The first cohort of the CDS was launched in 1997 and observed children every five years across three waves from 1997 to 2007 in order to study the dynamic process of early life human and social capital acquisition. In 2014, CDS began following a new cohort of children, conducting interviews with all eligible children in PSID families born or adopted since 1997 and their primary caregivers. Once children from CDS reach age 18 they are followed in the Transition into Adulthood Supplement (TAS), which started in 2005 and collects data biennially. The study captures data on developmental pathways and outcomes during the transition from childhood through young adulthood. As of 2017, all young adults age 18-28 are eligible for TAS, regardless of their participation in CDS.

**Measures:** Religious attendance was assessed with the question “How often did you go to religious services?” The participant could choose whether to report attendance as the number of times in either year, month, week, or day, depending on the participant’s frequency of religious attendance. For example, one participant could report attending 2 times per week and another could report 3 times per year (i.e., number of times and unit of time were coded as separate variables). Smoking was coded as current smoker vs. not. Heavy alcohol consumption was determined based on self-reported weekly intake and defined as 14 units or more men and 7 or more units for women. Physical inactivity was assessed based on self-reported frequency of moderate and vigorous physical activity, both items rated a scale from 0 to 4 (0=hardly ever or never; 1=one to three times a month; 2=once a week; 3=more than once a week; 4=every day) and defined as no moderate or vigorous activity (i.e., response of zero to both questions). Cannabis use was assessed as any use within last year. Depressive symptoms were assessed with 8 items of the CESD (response scale no/yes). Personal wellbeing was assessed with a 21-item version of Ryff’s personal wellbeing scale. Self-rated general health was reported on a 5-point scale (1=poor, 5=excellent). Body mass index was calculated from self-reported height and weight.

Study website: <https://psidonline.isr.umich.edu/default.aspx>

**Acknowledgements:** The collection of data used in this study was partly supported by the National Institutes of Health under grant number R01 HD069609 and R01 AG040213, and the National Science Foundation under award numbers SES 1157698 and 1623684.

**Data reference:** Panel Study of Income Dynamics, public use dataset. Produced and distributed by the Survey Research Center, Institute for Social Research, University of Michigan, Ann Arbor, MI, 2020.

| **Supplementary Table 1**. Descriptive statistics by study year. | | | | |  |
| --- | --- | --- | --- | --- | --- |
| **NLSY97** | | | | | |
| Year | Attendance | Smoking | Alcohol | Physical inactivity | Cannabis use |
| 2000 | 406 (100.0) | 405 (16.5) | 405 (7.4) | – | 405 (9.9) |
| 2001 | 693 (100.0) | 692 (18.9) | 691 (9.1) | – | 692 (11.4) |
| 2002 | 1028 (88.5) | 1027 (19.4) | 1027 (9.5) | – | 1023 (11.1) |
| 2003 | 1216 (80.3) | 1216 (21.6) | 1212 (13.1) | – | 1214 (11.1) |
| 2004 | 1368 (70.2) | 1367 (24.8) | 1356 (13.6) | – | 1351 (10.8) |
| 2005 | 1494 (63.9) | 1488 (23.1) | 1465 (12.0) | – | 1472 (10.6) |
| 2006 | 1603 (55.7) | 1599 (23.6) | 1580 (11.9) | – | 1572 (9.9) |
| 2007 | 1614 (52.5) | 1608 (24.3) | 1587 (11.1) | – | 1584 (9.0) |
| 2008 | 1605 (46.5) | 1598 (24.8) | 1573 (10.1) | – | 1576 (9.5) |
| 2009 | 1512 (39.1) | 1504 (23.5) | 1497 (10.7) | – | 1491 (9.4) |
| 2010 | 1364 (36.6) | 1360 (23.8) | 1339 (11.3) | – | 1339 (10.2) |
| 2011 | 1189 (29.4) | 1186 (23.9) | 1171 (11.1) | – | 1167 (10.5) |
| 2015 | 432 (25.9) | 429 (22.4) | 426 (10.1) | – | 426 (9.9) |
| 2017 | 389 (0.0) | – | – | – | – |
| Note: Values are numbers of person-observations per study wave. The values in parenthesis gives the percentage of person-observations endorsing the variable (0=no, 1=yes). | | | | | |
|  |  |  |  |  |  |
| Year | Depressive symptoms | Self-rated health | Body mass index | Psychological wellbeing |  |
| 2000 | 406 (0.9) | 405 (4.2) | 394 (24.2) | – |  |
| 2001 | – | 693 (4.1) | 674 (24.8) | – |  |
| 2002 | 1028 (0.9) | 1027 (4.0) | 948 (25.2) | – |  |
| 2003 | – | 1216 (4.0) | 1117 (25.9) | – |  |
| 2004 | 1364 (0.9) | 1367 (4.0) | 1308 (26.1) | – |  |
| 2005 | – | 1494 (3.9) | 1432 (26.5) | – |  |
| 2006 | 1602 (0.9) | 1602 (3.9) | 1534 (26.9) | – |  |
| 2007 | – | 1614 (3.9) | 1554 (27.3) | – |  |
| 2008 | 1604 (0.9) | 1604 (3.8) | 1556 (27.6) | – |  |
| 2009 | – | 1512 (3.8) | 1463 (27.9) | – |  |
| 2010 | 1362 (0.9) | 1363 (3.7) | 1311 (28.0) | – |  |
| 2011 | – | 1189 (3.7) | 1155 (28.4) | – |  |
| 2015 | 432 (0.8) | 432 (3.6) | 411 (29.0) | – |  |
| 2017 | – | 389 (3.5) | 380 (29.6) | – |  |
| Note: Values are numbers of person-observations per study wave. The values in parenthesis gives the mean value of the variable. | | | | | |

*(Supplementary Table 1 continues …)*

| **NLSY-YA** | | | | | |
| --- | --- | --- | --- | --- | --- |
| Year | Attendance | Smoking | Alcohol | Physical inactivity | Cannabis use |
| 1996 | 20 (100.0) | 7 (14.3) | 14 (0.0) | – | 15 (6.7) |
| 1998 | 86 (100.0) | 76 (17.1) | 66 (1.5) | – | 76 (17.1) |
| 2000 | 236 (97.9) | 236 (19.9) | 214 (1.4) | – | 234 (9.4) |
| 2002 | 423 (91.7) | 423 (20.6) | 375 (4.3) | – | 422 (15.6) |
| 2004 | 599 (82.6) | 598 (21.9) | 528 (5.5) | 599 (23.5) | 595 (11.3) |
| 2006 | 769 (68.3) | 764 (24.2) | 658 (8.1) | 768 (24.0) | 751 (14.4) |
| 2008 | 890 (65.5) | 885 (22.3) | 769 (7.4) | 889 (26.2) | 869 (15.1) |
| 2010 | 874 (61.8) | 874 (21.6) | 761 (8.2) | 872 (23.2) | 858 (16.1) |
| 2012 | 770 (51.3) | 768 (21.2) | 669 (7.8) | 770 (22.7) | 762 (17.5) |
| 2014 | 569 (36.0) | 568 (20.1) | 510 (9.8) | 567 (21.9) | 566 (19.8) |
| 2016 | 390 (0.0) | 390 (18.7) | 335 (7.8) | 389 (23.9) | 386 (21.2) |
| Note: Values are numbers of person-observations per study wave. The values in parenthesis gives the percentage of person-observations endorsing the variable (0=no, 1=yes). | | | | | |
|  |  |  |  |  |  |
| Year | Depressive symptoms | Self-rated health | Body mass index | Psychological wellbeing |  |
| 1996 | 20 (0.6) | 20 (3.9) | 19 (25.2) | – |  |
| 1998 | 86 (0.7) | 86 (4.0) | 83 (25.5) | – |  |
| 2000 | 57 (0.6) | 236 (3.8) | 235 (25.9) | – |  |
| 2002 | 58 (0.7) | 423 (3.8) | 413 (26.2) | – |  |
| 2004 | 598 (0.7) | 599 (3.8) | 594 (26.4) | – |  |
| 2006 | 769 (0.7) | 769 (3.8) | 763 (26.8) | – |  |
| 2008 | 888 (0.7) | 890 (3.8) | 882 (26.8) | – |  |
| 2010 | 874 (0.6) | 874 (3.8) | 865 (26.8) | – |  |
| 2012 | 473 (0.6) | 770 (3.8) | 762 (27.2) | – |  |
| 2014 | 259 (0.5) | 569 (3.6) | 565 (27.7) | – |  |
| 2016 | 218 (0.6) | 390 (3.6) | 387 (28.4) | – |  |
| Note: Values are numbers of person-observations per study wave. The values in parenthesis gives the mean value of the variable. | | | | | |

*(Supplementary Table 1 continues …)*

| **PSID-TA** | | | | | |
| --- | --- | --- | --- | --- | --- |
| Year | Attendance | Smoking | Alcohol | Physical inactivity | Cannabis use |
| 2005 | 81 (100.0) | 81 (18.5) | 80 (2.5) | 81 (18.5) | 81 (21.0) |
| 2007 | 146 (100.0) | 145 (24.8) | 146 (7.5) | 146 (35.6) | 146 (21.9) |
| 2009 | 210 (77.6) | 210 (21.4) | 209 (5.7) | 210 (45.7) | 210 (24.8) |
| 2011 | 242 (61.6) | 242 (20.7) | 241 (5.8) | 242 (60.3) | 242 (28.9) |
| 2013 | 223 (57.0) | 223 (20.6) | 222 (5.9) | 223 (57.9) | 223 (27.4) |
| 2015 | 160 (41.3) | 160 (20.6) | 160 (3.8) | 160 (66.9) | 160 (28.8) |
| 2017 | 109 (0.0) | 109 (21.1) | 108 (4.6) | 109 (19.3) | 109 (41.3) |
| Note: Values are numbers of person-observations per study wave. The values in parenthesis gives the percentage of person-observations endorsing the variable (0=no, 1=yes). | | | | | |
|  |  |  |  |  |  |
| Year | Depressive symptoms | Self-rated health | Body mass index | Psychological wellbeing |  |
| 2005 | 81 (0.6) | 81 (4.1) | 80 (24.3) | 81 (5.2) |  |
| 2007 | 146 (0.7) | 146 (3.9) | 145 (25.6) | 146 (5.2) |  |
| 2009 | 210 (0.6) | 210 (3.9) | 207 (26.0) | 210 (5.2) |  |
| 2011 | 242 (0.6) | 242 (3.8) | 240 (26.4) | 242 (5.1) |  |
| 2013 | 223 (0.6) | 223 (3.8) | 221 (27.5) | 223 (5.0) |  |
| 2015 | 160 (0.6) | 160 (3.6) | 158 (28.5) | 160 (5.0) |  |
| 2017 | 109 (0.6) | 109 (3.6) | 106 (29.4) | 109 (5.1) |  |
| Note: Values are numbers of person-observations per study wave. The values in parenthesis gives the mean value of the variable. | | | | | |

*(Supplementary Table 1 continues …)*

| **HRS** | | | | | |
| --- | --- | --- | --- | --- | --- |
| Year | Attendance | Smoking | Alcohol | Physical inactivity | Cannabis use |
| 2004 | 1811 (100.0) | 1798 (12.2) | 1515 (1.2) | – | – |
| 2006 | 2261 (100.0) | 2245 (11.4) | 1871 (1.2) | 2261 (39.7) | – |
| 2008 | 2469 (80.4) | 2453 (11.4) | 2092 (1.3) | 2469 (43.2) | – |
| 2010 | 2421 (64.3) | 2404 (10.8) | 2044 (1.2) | 2419 (48.6) | – |
| 2012 | 2218 (52.9) | 2205 (10.8) | 1858 (1.3) | 2218 (49.4) | – |
| 2014 | 1800 (42.4) | 978 (17.9) | 1488 (1.6) | 1797 (51.4) | – |
| 2016 | 1290 (24.8) | 711 (16.9) | 1085 (1.3) | 1287 (52.9) | – |
| 2018 | 791 (0.0) | 437 (15.1) | 642 (1.4) | 791 (57.5) | – |
| Note: Values are numbers of person-observations per study wave. The values in parenthesis gives the percentage of person-observations endorsing the variable (0=no, 1=yes). | | | | | |
|  |  |  |  |  |  |
| Year | Depressive symptoms | Self-rated health | Body mass index | Psychological wellbeing |  |
| 2004 | 1715 (1.5) | 1810 (3.1) | 1782 (27.8) | – |  |
| 2006 | 2162 (1.6) | 2257 (3.0) | 2228 (28.2) | 970 (4.1) |  |
| 2008 | 2317 (1.6) | 2467 (2.9) | 2428 (28.3) | 1001 (4.5) |  |
| 2010 | 2198 (1.7) | 2419 (2.9) | 2378 (28.2) | 932 (4.5) |  |
| 2012 | 2009 (1.7) | 2216 (2.9) | 2187 (28.1) | 782 (4.4) |  |
| 2014 | 1617 (1.8) | 1799 (2.8) | 915 (28.5) | 723 (4.4) |  |
| 2016 | 1140 (1.9) | 1287 (2.8) | 588 (28.4) | 386 (4.3) |  |
| 2018 | 685 (2.6) | 790 (2.7) | 422 (28.2) | 256 (4.3) |  |
| Note: Values are numbers of person-observations per study wave. The values in parenthesis gives the mean value of the variable. | | | | | |

| **Supplementary Table 2.** Meta-analytic estimates for the piecewise regerssion models for trends before and after change from active to inactive religious attendance. | | | |
| --- | --- | --- | --- |
| Variable | Before | After | p(int) |
| **Main analysis** |  |  |  |
| Smoking | 0.11 (0.04, 0.17) | -0.01 (-0.07, 0.05) |  |
| Alcohol | 0.17 (0.07, 0.27) | 0.05 (-0.03, 0.13) | 0.07 |
| Physical inactivity | 0.07 (-0.05, 0.20) | 0.02 (-0.09, 0.13) | 0.58 |
| Cannabis use | 0.09 (0.01, 0.17) | 0.14 (0.10, 0.19) | 0.25 |
| Depressive symptoms | 0.01 (-0.01, 0.03) | 0.01 (-0.02, 0.03) | 0.82 |
| Personal wellbeing | 0.01 (-0.03, 0.05) | -0.01 (-0.03, 0.00) | 0.21 |
| Self-rated health | -0.02 (-0.04, 0.01) | -0.02 (-0.05, 0.00) | 0.84 |
| Body mass index | 0.12 (0.10, 0.15) | 0.08 (0.05, 0.10) | 0.01 |
| **Fixed-effect** |  |  |  |
| Smoking | 0.11 (-0.12, 0.35) | -0.08 (-0.28, 0.12) | 0.22 |
| Alcohol | 0.09 (-0.04, 0.23) | 0.06 (-0.10, 0.21) | 0.71 |
| Physical inactivity | 0.04 (-0.17, 0.26) | 0.10 (-0.06, 0.27) | 0.65 |
| Cannabis use | -0.09 (-0.26, 0.08) | -0.03 (-0.20, 0.13) | 0.65 |
| Depressive symptoms | 0.00 (-0.09, 0.09) | 0.01 (-0.02, 0.05) | 0.82 |
| Personal wellbeing | 0.04 (-0.13, 0.22) | -0.04 (-0.06, -0.02) | 0.36 |
| Self-rated health | 0.01 (-0.02, 0.04) | 0.00 (-0.01, 0.02) | 0.74 |
| Body mass index | 0.30 (0.13, 0.47) | 0.15 (-0.05, 0.36) | 0.29 |
| **Weekly vs. less often** |  |  |  |
| Smoking | 0.15 (0.02, 0.27) | 0.02 (-0.04, 0.08) | 0.07 |
| Alcohol | 0.24 (0.04, 0.44) | 0.09 (-0.05, 0.23) | 0.23 |
| Physical inactivity | 0.06 (-0.06, 0.19) | 0.03 (-0.08, 0.13) | 0.64 |
| Cannabis use | 0.19 (0.09, 0.29) | 0.21 (0.09, 0.34) | 0.79 |
| Depressive symptoms | 0.01 (-0.01, 0.03) | 0.02 (-0.01, 0.05) | 0.60 |
| Personal wellbeing | 0.02 (-0.01, 0.05) | -0.03 (-0.06, 0.01) | 0.07 |
| Self-rated health | -0.02 (-0.04, 0.01) | -0.03 (-0.05, -0.01) | 0.40 |
| Body mass index | 0.11 (0.06, 0.15) | 0.08 (0.04, 0.11) | 0.26 |
| Note: Values are regression coefficients of piecewise linear or logistic regression models, adjusted for age, sex, race/ethnicity, education, and marital status (maximum n=37721 person-observations of 6592 individuals). p(int) = statisticial significance of the difference between before vs after. | | | |

| **Supplementary Table 3**. Health trajectories before and after the change from inactive to active religious attendance, additionally fitted with fixed-effect regression and with weekly vs less than weekly attendance. | | | |
| --- | --- | --- | --- |
|  | Before | After | p(int) |
| **Main analysis** |  |  |  |
| Smoking | -0.06 (-0.10, -0.02) | -0.19 (-0.28, -0.09) | 0.02 |
| Alcohol | -0.08 (-0.11, -0.04) | -0.09 (-0.24, 0.07) | 0.90 |
| Physical inactivity | 0.00 (-0.07, 0.07) | -0.03 (-0.10, 0.05) | 0.56 |
| Cannabis use | -0.06 (-0.12, 0.00) | -0.05 (-0.23, 0.12) | 0.98 |
| Depressive symptoms | 0.01 (0.00, 0.02) | -0.01 (-0.03, 0.01) | 0.11 |
| Personal wellbeing | 0.02 (-0.01, 0.06) | 0.01 (0.00, 0.02) | 0.43 |
| Self-rated health | -0.01 (-0.02, 0.00) | 0.01 (0.00, 0.02) | 0.00 |
| Body mass index | 0.14 (0.09, 0.20) | 0.10 (0.05, 0.15) | 0.26 |
| **Fixed-effect** |  |  |  |
| Smoking | 0.09 (-0.27, 0.45) | -0.11 (-0.27, 0.04) | 0.31 |
| Alcohol | 0.01 (-0.10, 0.11) | -0.05 (-0.16, 0.06) | 0.47 |
| Physical inactivity | 0.06 (0.02, 0.11) | 0.10 (-0.13, 0.33) | 0.73 |
| Cannabis use | -0.24 (-0.36, -0.13) | -0.31 (-0.43, -0.19) | 0.41 |
| Depressive symptoms | 0.01 (-0.06, 0.07) | 0.00 (-0.01, 0.01) | 0.81 |
| Personal wellbeing | 0.06 (-0.15, 0.28) | -0.01 (-0.11, 0.10) | 0.57 |
| Self-rated health | -0.02 (-0.04, 0.00) | 0.01 (0.00, 0.02) | 0.00 |
| Body mass index | 0.42 (0.23, 0.61) | 0.27 (0.03, 0.50) | 0.32 |
| **Weekly vs. less often** |  |  |  |
| Smoking | -0.03 (-0.07, 0.01) | -0.25 (-0.38, -0.12) | 0.00 |
| Alcohol | -0.06 (-0.10, -0.03) | -0.05 (-0.22, 0.12) | 0.88 |
| Physical inactivity | 0.03 (-0.06, 0.11) | -0.02 (-0.12, 0.07) | 0.43 |
| Cannabis use | -0.08 (-0.13, -0.03) | -0.17 (-0.24, -0.09) | 0.06 |
| Depressive symptoms | 0.00 (-0.02, 0.02) | -0.02 (-0.05, 0.01) | 0.22 |
| Personal wellbeing | 0.03 (-0.01, 0.07) | 0.00 (-0.01, 0.02) | 0.32 |
| Self-rated health | -0.01 (-0.02, 0.00) | 0.01 (-0.01, 0.02) | 0.09 |
| Body mass index | 0.13 (0.07, 0.19) | 0.09 (0.05, 0.14) | 0.32 |
| Note: Values are regression coefficients of piecewise linear or logistic regression models, adjusted for age, sex, race/ethnicity, education, and marital status (maximum n=34924 person-observations of 5852 individuals). p(int) = statisticial significance of the difference between before vs after. | | | |

**Supplementary Figure 1**. Odds ratios of smoking associated with time before vs after the change from active to inactive religious attendance (in years). Reference is the index year, that is, the last study wave of the participant’s active attendance over the follow-up period.

**Supplementary Figure 2**. Odds ratios of heavy alcohol consumption associated with time before vs after the change from active to inactive religious attendance (in years). Reference is the index year, that is, the last study wave of the participant’s active attendance over the follow-up period.

**Supplementary Figure 3**. Odds ratios of physical inactivity associated with time before vs after the change from active to inactive religious attendance (in years). Reference is the index year, that is, the last study wave of the participant’s active attendance over the follow-up period.

**Supplementary Figure 4**. Odds ratios of cannabis use associated with time before vs after the change from active to inactive religious attendance (in years). Reference is the index year, that is, the last study wave of the participant’s active attendance over the follow-up period.

**Supplementary Figure 5**. Trajectory slopes of smoking associated with time before vs after the change from active to inactive religious attendance (piecewise regression).

**Supplementary Figure 6**. Trajectory slopes of heavy alcohol consumption associated with time before vs after the change from active to inactive religious attendance (piecewise regression).

**Supplementary Figure 7**. Trajectory slopes of physical inactivity associated with time before vs after the change from active to inactive religious attendance (piecewise regression).

**Supplementary Figure 8**. Trajectory slopes of cannabis use associated with time before vs after the change from active to inactive religious attendance (piecewise regression).

**Supplementary Figure 9**. Health trajectories associated with time before vs after the change from active to inactive religious attendance (in years) when estimated with fixed-effect regression.

**Supplementary Figure 10**. Health trajectories associated with time before vs after the change from active to inactive religious attendance (in years) when comparing “weekly” vs “less often” religious attendance.

**Supplementary Figure 11**. Standardized mean differences in depressive symptoms associated with time before vs after the change from active to inactive religious attendance (in years). Reference is the index year, that is, the last study wave of the participant’s active attendance over the follow-up period.

**Supplementary Figure 12.** Standardized mean differences in personal wellbeing associated with time before vs after the change from active to inactive religious attendance (in years). Reference is the index year, that is, the last study wave of the participant’s active attendance over the follow-up period.

**Supplementary Figure 13.** Mean differences in self-rated health associated with time before vs after the change from active to inactive religious attendance (in years). Reference is the index year, that is, the last study wave of the participant’s active attendance over the follow-up period.

**Supplementary Figure 14**. Mean differences in depressive symptoms associated with time before vs after the change from active to inactive religious attendance (in years). Reference is the index year, that is, the last study wave of the participant’s active attendance over the follow-up period.

**Supplementary Figure 15.** Trajectory slopes of depressive symptoms associated with time before vs after the change from active to inactive religious attendance (piecewise regression).

**Supplementary Figure 16.** Trajectory slopes of personal wellbeing associated with time before vs after the change from active to inactive religious attendance (piecewise regression).

**Supplementary Figure 17**. Trajectory slopes of self-rated health associated with time before vs after the change from active to inactive religious attendance (piecewise regression).

**Supplementary Figure 18**. Trajectory slopes of body mass index associated with time before vs after the change from active to inactive religious attendance (piecewise regression).

**Supplementary Figure 19**. Health trajectories associated with time before vs after the change from active to inactive religious attendance (in years) when estimated with fixed-effect regression.

**Supplementary Figure 20**. Health trajectories associated with time before vs after the change from active to inactive religious attendance (in years) when comparing “weekly” vs “less often” religious attendance.

**Supplementary Figure 21**. Health trajectories associated with time before vs after an index wave that was selected at random for all the participants of the cohort studies.

**Supplementary Figure 22**. Health trajectories associated with time before vs after an index wave that was selected at random for all the participants of the cohort studies.

**Figure 23**. Log odds ratios (top two rows) and mean differences (bottom two rows) in health outcomes before and after the change from active to inactive religious attendance (monthly or more vs. less), pooled across the four cohort studies with random-effects meta-analysis. The reference category is zero, the index wave of the participant’s last measurement time of active religious attendance during the follow-up time. Error bars are 95% confidence intervals. p(int) = statistical significance for the difference in fitted lines before vs after index wave. Values are standardized scores (standard deviation=1) for depressive symptoms and personal wellbeing, and non-standardized scores for self-rated health and body mass index.

**Supplementary Figure 24**. Odds of smoking associated with time before vs after the change from inactive to active religious attendance (in years). Reference is the index year, that is, the last study wave of the participant’s active attendance over the follow-up period.

**Supplementary Figure 25**. Odds of heavy alcohol consumption associated with time before vs after the change from inactive to active religious attendance (in years). Reference is the index year, that is, the last study wave of the participant’s active attendance over the follow-up period.

**Supplementary Figure 26**. Odds of physical inactivity associated with time before vs after the change from inactive to active religious attendance (in years). Reference is the index year, that is, the last study wave of the participant’s active attendance over the follow-up period.

**Supplementary Figure 27**. Odds of cannabis use associated with time before vs after the change from inactive to active religious attendance (in years). Reference is the index year, that is, the last study wave of the participant’s active attendance over the follow-up period.

**Supplementary Figure 28.** Standardized mean differences in depressive symptoms associated with time before vs after the change from inactive to active religious attendance (in years). Reference is the index year, that is, the last study wave of the participant’s active attendance over the follow-up period.

**Supplementary Figure 29.** Standardized mean differences in personal wellbeing associated with time before vs after the change from inactive to active religious attendance (in years). Reference is the index year, that is, the last study wave of the participant’s active attendance over the follow-up period.

**Supplementary Figure 30**. Mean differences in self-rated health associated with time before vs after the change from inactive to active religious attendance (in years). Reference is the index year, that is, the last study wave of the participant’s active attendance over the follow-up period.

**Supplementary Figure 31.** Mean differences in body mass index associated with time before vs after the change from inactive to active religious attendance (in years). Reference is the index year, that is, the last study wave of the participant’s active attendance over the follow-up period.

**Supplementary Figure 32.** Health trajectories associated with time before vs after the change from inactive to active religious attendance (in years) when estimated with fixed-effect regression.

**Supplementary Figure 33.** Health trajectories associated with time before vs after the change from active to inactive religious attendance (in years) when comparing “weekly” vs “less often” religious attendance.
